# Poster Session II - A273 TWSG1 VARIATION IDENTIFIES A USTEKINUMAB-RESPONSE PHENOTYPE IN PAITNETS WITH INFLAMMATORY BOWEL DISEASE

**DOI:** 10.1093/jcag/gwaf042.273

**Published:** 2026-02-13

**Authors:** M Alkhalifa, T Ponich, J Gregor, M Beaton, K McIntosh, R Khanna, B Yan, R Kim, A Wilson

**Affiliations:** Gastroenterology, Western University, London, ON, Canada; Gastroenterology, Western University, London, ON, Canada; Gastroenterology, Western University, London, ON, Canada; Gastroenterology, Western University, London, ON, Canada; Gastroenterology, Western University, London, ON, Canada; Gastroenterology, Western University, London, ON, Canada; Gastroenterology, Western University, London, ON, Canada; Gastroenterology, Western University, London, ON, Canada; Medicine, Western University, London, ON, Canada

## Abstract

**Background:**

Blocking Th17-driven inflammation by targeting interleukin-23 cytokines has become central to inflammatory bowel disease therapeutics. Ustekinumab, a human monoclonal IgG1 antibody, blocks the shared p40 subunit of IL-12 and IL-23. Up to 50% of patients do not respond to ustekinumab which suggests the need for more effective patient selection. A conference abstract by Hart et.al. (2017), identified that a genetic variation in the twisted gastrulation protein homolog-1 gene (TWSG1 rS7242593) was linked to early clinical disease remission in patients exposed to Ustekinumab enrolled in the UNITI clinical trials. This data suggests a potential role for this genetic variation to predict patient’s likelihood of response to Ustekinumab; however, it has not been validated in an independent cohort.

**Aims:**

To confirm if a single nucleotide variation (SNV) in the TWSG1 gene is associated with early disease remission in IBD patients on Ustekinumab as well as other important clinical outcomes.

**Methods:**

A retrospective cohort study was conducted in IBD patients on Ustekinumab.All participants underwent screening for the single nucleotide variation (SNV) rs7242593 in the TWSG1 gene and were assessed for clinical disease remission by Harvey-Bradshaw index or partial Mayo score at 3 months and at 12 months. Ustekinumab dose and treatment duration were additionally assessed.

**Results:**

A total of 125 participants with IBD, seen at a tertiary care centre in London, Canada, were included in the final analysis (wildtype genotype, CC, n = 108; variant genotype, CT, n = 17).The median follow-up time from the date of IBD diagnosis was 8 years (interquartile range, IQR=9 years). Baseline characteristics were similar between wild type and variant genotype groups. Participants in the variant genotype group were more likely to achieve clinical remission at 3-months (odds ratio, OR 23.11, 95% confidence interval, CI = 3.92-449.40, adjusted p = 0.0043), at 12-months (OR = 17.75, 95%CI=2.42-381.0, adjusted p = 0.016) on standard Ustekinumab dosing and less likely to discontinue therapy during the follow up period (hazard ratio=4.20, 95%CI=1.94-9.09, p = 0.02). Ustekinumab dose escalation was not associated with disease remission at 3 months (OR = 0.30, 95%Cl:0.12-0.75, adjusted p < 0.0001) or 12 months (OR = 0.13, 95%CI=0.04-0.33, p < 0.0001).

**Conclusions:**

SNV (rs7242593) in TWSG1 was associated with early and persistent clinical remission in Ustekinumab-exposed IBD patients. Wild-type participants who did not achieve remission could not be rescued with high dose treatment. This SNV may also serve as a useful molecular marker of patients who do not benefit from Ustekinumab dose accelerations.

**Funding Agencies:**

None

